# Socio-economic factors linked with mental health during the recession: a multilevel analysis

**DOI:** 10.1186/s12939-017-0518-x

**Published:** 2017-03-06

**Authors:** Isabel Ruiz-Pérez, Clara Bermúdez-Tamayo, Miguel Rodríguez-Barranco

**Affiliations:** 1 Escuela Andaluza de Salud Pública, Campus Universitario de Cartuja s/n, Granada, 18080 Spain; 2 CIBER Epidemiology and Public Health (CIBERESP), Barcelona, Spain; 3 Biosanitary Institute of Granada (ibs.GRANADA), Granada, Spain

**Keywords:** Mental Health, Health Inequalities, Financial crisis, Recession, Socio-economic indicators

## Abstract

**Background:**

Periods of financial crisis are associated with higher psychological stress among the population and greater use of mental health services. The objective is to analyse contextual factors associated with mental health among the Spanish population during the recession.

**Methodology:**

Cross-sectional, descriptive study of two periods: before the recession (2006) and after therecession *(2011-2012)*. The study population comprised individuals aged 16+ years old, polled for the National Health Survey. There were 25,234 subjects (2006) and 20,754 subjects (2012). The dependent variable was psychic morbidity. Independent variables: 1) socio-demographic (age, socio-professional class, level of education, nationality, employment situation, marital status), 2) psycho-social (social support) and 3) financial (GDP per capita, risk of poverty, income per capita per household), public welfare services (health spending per capita), labour market (employment and unemployment rates, percentage of temporary workers). Multilevel logistic regression models with mixed effects were constructed to determine change in psychic morbidity according to the variables studied.

**Results:**

The macroeconomic variables associated with worse mental health for both males and females were lower health spending per capita and percentage of temporary workers. *Among women, the risk of poor mental health increased 6% for each 100€ decrease in healthcare spending per capita. Among men, the risk of poor mental health decreased 8% for each 5-percentage point increase in temporary workers*.

**Conclusions:**

Higher rates of precarious employment in a region have a negative effect on people’s mental health; likewise lower health spending per capita. Policies during periods of recession should focus on support and improved conditions for vulnerable groups such as temporary workers. Healthcare cutbacks should be avoided in order to prevent increased prevalence of poor mental health.

**Electronic supplementary material:**

The online version of this article (doi:10.1186/s12939-017-0518-x) contains supplementary material, which is available to authorized users.

## Background

The effects of financial crises on health have been studied for decades. The evidence suggests that recessions have damaging effects on many health indicators, *particularly mortality and suicide* [1]*. There is also evidence that financial crises can have some positive effects on health (e.g. fewer workplace accidents or less tobacco consumption), although in general results are more heterogenous* [2]*. Furthermore, periods of financial* crisis are associated with higher psychological stress among the population and greater use of mental health services [3, 4]. Increased levels of anxiety and depression are equally recorded [5]. In turn, these conditions are associated with an increase in the number of attempted suicides and premature deaths due to episodes of violence and suicide [6, 7] and increased consumption of alcohol [8].

However, the effects of an economic downturn do not have the same impact on all individuals and all countries; sex, age, level of education, marital status, size of household, employment, income, belief systems and social relationships are individual factors which have a bearing on better or worse resilience [9]. And socio-economic factors can also play a part in this impact. Analysis of the policies implemented by some countries during times of economic crisis reveals the link between these policies and impact on mental health among the population [10–12]. Austerity measures such as the massive cutbacks made as a result of the crisis in different European countries have had a harmful effect on mental health [11]. Precisely when individuals may require more care due to mental health problems, cutbacks in the healthcare sector may lead to reduced services for prevention, early detection and treatment of mental health problems. In this respect, vulnerable groups - people in financial difficulty and people with health issues - would be at higher risk [13]. The meta-analysis by Paul and Moser [14] showed that the negative effect of unemployment on mental health was more pronounced in countries with a low level of economic development, unequal distribution of income or weak unemployment benefit systems.

The effect of contextual factors has been noted in highly diverse geographical areas distant from Spain such as Asia, where the economic crisis appears to have had a lower impact on health in Malaysia than in Thailand or Indonesia. Unlike its neighbours, Malaysia rejected World Bank advice to make cutbacks in healthcare spending [12].

Spain has stood out as one of the countries most severely affected by the so-called great recession [15], one of the most overwhelming effects of which is unemployment [15–17]. To analyse the impact on health of the crisis in Spain, two particularities must be taken into account: on the one hand, the healthcare system provides almost universal coverage and on the other, there are differences between regions as a result of political decentralisation. An example of this is the spending gap per inhabitant between the regions with the highest and lowest spending, reaching 62% in 2014 [18]. As regards social protection (retirement pension, sickness or disability benefit, unemployment benefit, measures to protect families and prevent social exclusion), this gap was 87% [18]. A recent study detected major differences in austerity measures during the recession [19]; whilst in the Basque Country policies for austerity and privatisation were almost non-existent, the trend in other regions such as La Rioja, Madrid and the Balearic Islands was clearly in the opposite direction.

This reality may determine variations in the impact of the recession depending on the region where people live, as a result of how different Autonomous Community governments have responded to the recession. Studies on the impact on mental health of contextual factors between regions in the same country are limited [9–14, 20] and we consider that looking at regions in a single country facilitates comparison given similarities in the population as regards culture, values and belief systems.

Various articles have addressed the impact of socio-economic crises on mental health in Spain [3–5, 8, 13, 15–18, 21–25]. They have focused only on analysing the effect of *individual factors. But in addition to these individual variables there are contextual variables which can either lessen or intensify the adverse effects of the crisis, amongst which are the variables relating to the political and institutional context, such as economic indicators, public welfare services indicators and labour market indicators.*

The impact of the crisis on the health of the population could be lessened or intensified by policies, affecting the financial security and social conditions of families [1].

The aim of this study is to analyse the socio-economic factors impacting on mental health during the recession in Spain.

## Methods

### Design

Cross-sectional descriptive study of two periods: before the recession (2006) and after the recession *(2011-2012).*

### Study population

Individuals aged 16+ years old, resident in Spain, polled for the National Health Survey in 2006 and 2012. There were 25,234 subjects in 2006 and 20,754 subjects in 2012.

### Variables

#### Dependent

Psychic morbidity measured through self-referred poor mental health: yes (GHQ > = 3)/no (GHQ < 3). According to the Goldberg Health Questionnaire, 12 items (GHQ-12), adapted and validated in our environment.

#### Individual independent

- Socio-demographic variables: a) axes of social inequality: age, socio-professional class, level of education (low, medium or high, as per ISCED International Standard Classification of Education). Low level equates to no schooling or primary education, medium level equates to secondary education and mid-grade vocational training, and high level equates to advanced vocational training and university qualifications, nationality; b) other: employment situation, marital status. Social class has been determined based on current or most recent professional occupation according to National Occupation Classification CNO-2011.

Psycho-social variables: social support (emotional and personal support collated by means of the Duke-UNC Functional Social Support Questionnaire).

### Contextual independent

*The contextual variables were selected on the basis of their availability for the years analyzed and the degree of disaggregation by region* *(Additional file* *1* *)*. *The geographical unit of analysis is based on NUTS-2 regions of EUROSTAT (called Autonomous Communities in Spain).*

- *Economic indicators:* Gross Domestic Product (GDP) per capita at current prices (ratio to the Spain average x100), risk of poverty (%), income per capita per household (ratio to the Spain average x100).
- *Public welfare services indicators:* healthcare spending per capita (euros).
- *Labour market indicators: employment rate (per 100 person-year), unemployment rate (per 100 person-year), percentage of temporary workers (%).*

### Data sources

Data on individuals were obtained from the Spanish National Health Survey (ENSE) for 2006 and 2012. This is a cross-sectional and population-based survey by the National Institute of Statistics (INE) working with the Ministry of Health, Social Services and Equality, which collates health information by household. A tri-stage sampling method was used, stratified into census sections, family dwellings and persons, and the data were gathered through computer-assisted personal interviews.

*To calculate socio-economic indicators, we used data from the National Institute of Statistics (GDP per capita, income per capita per household and risk of poverty)* [26, 27]*; Eurostat (employment and unemployment rates, percentage of temporary workers)* [28]*; and BBVA Foundation (healthcare spending per capita)* [29].

### Data analysis

All the analyses were performed by sex (male and female) and for the total population. Prevalence was calculated for the psychic morbidity variable and the independent proportions comparison test was applied to compare significant changes. The Chi-square test was used to compare determinant bivariates between the two periods.

Two multilevel logistic regression models with random effect were constructed to determine change in psychic morbidity according to individual and contextual variables respectively. In the first model, the study period and predictor variables at the individual and socio-economic level were included, and intercepts at the NUTS-2 region level were included as random effect. In the second model, contextual variables were included individually (in order to avoid collinearity) and adjusted for individual characteristics, and *intercepts at the NUTS-2 region level were included as random effect.*

In all models, whether the differences are significant was assessed by using the Wald test for each predictor. Correction of the clustered robust variance was done by the observed information matrix (OIM). *The magnitude of effects is measured by the odds ratio (OR) and 95% confidence interval, and a significance level of 0.05 will be set for hypothesis checking. In the indicator models for the macroeconomic context, the magnitude of association was expressed for a change of approximately one standard deviation of the context variable analysed.*

Statistical analyses were performed using Stata software (StataCorp., TX).

## Results

Between 2006 and 2011-2012, the pattern of psychic morbidity differed between men and women.

Among men, poor mental health has increased significantly in the 30 - 34 age group (14.2%-17.0%) and in the 45 - 59 age group (16.1%-19.9%), also among single men (14.4%-17.2%) and married men (14.5-16.7%), men with a low level of education (17.5%-19.8%) and normal social support (14.6%-16.8%). Country of origin was not found to have any link to differences in prevalence of poor mental health, since this was significant for Spaniards and foreigners alike. Nor was any link found between socio-professional class and differences in prevalence of psychic morbidity (Table 1).

**Table 1:**
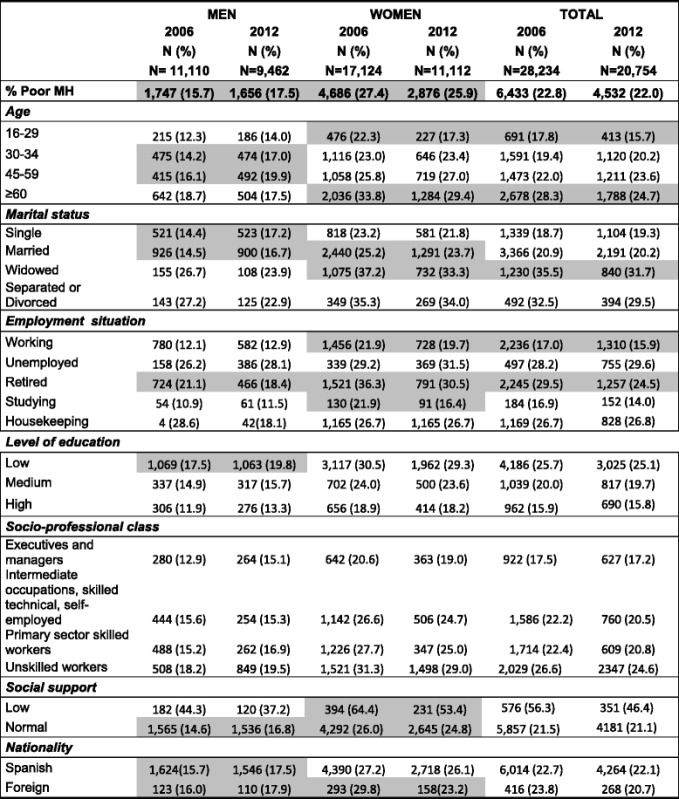
Prevalence of poor mental health (according to individual characteristics), 2006 and 2012

Values in grey*: p < 0.05*

Among women, groups which showed significant differences in mental health between 2006 and 2012 were the 16 - 29 age group (drop from 22.3% to 17.3%) and the over-60 age group (drop from 33.8% to 29.4%). Married women (25.3%-23.7%) and widows (37.2%-33.3%) also showed a significant decrease in the prevalence of poor mental health, similarly to working women (21.9%-19.7), retired women (36.3%-30.5%) and women studying (21.9%-16.4%).

In accordance with the first multilevel logistic regression model (Table 2) for men, widowers (OR: 1.45 CI 95%: 1.27-1.55) presented a higher risk of poor health compared with single men, as did separated or divorced men (OR: 1.54 CI 95%: 1.33-1.78). By contrast, married men (OR: 1.97 CI 95%: 0.91-0.798) presented a lower risk of psychic morbidity than single men. As regards employment situation, unemployed men presented a higher risk of psychic morbidity in comparison to working men (OR: 1.81; CI 95%: 1.67-1.98) and retired men (OR: 1.23; CI 95%: 1.12-1.35). Lastly, a link was found between better social support and lower risk of psychic morbidity.

**Table 2:** Multi-level logistic regression model with random effects at NUTS-2 regions level according to individual variables for poor mental health (GHQ ≥ 3)

Independent variable	Men			Women		
	OR	95% CI	*p*-value	OR	95% CI	*p*-value
Year						
2006	1	-	-	1	-	-
2012	1.03	(0.99 - 1.09)	0.202	1.01	(0.97 - 1.11)	0.500
Age group						
16-29	1	-	-	1	-	-
30-44	1.01	(0.91 - 1.11)	0.893	1.01	(0.91 - 1.21)	0.893
45-59	0.82	(0.74 - 0.92)	0.071	1.11	(0.74 - 0.92)	0.061
> =60	0.60	(0.54 - 0.7)	0.099	0.61	(0.54 - 0.7)	0.062
Marital status						
Single	1	-	-	1	-	-
Married	0.97	(0.91 - 0.98)	0.042	1.02	(0.91 - 1.04)	0.459
Widowed	1.45	(1.27 - 1.55)	<0.001	1.40	(1.24 - 1.57)	<0.001
Separated or Divorced	1.54	(1.33 - 1.78)	<0.001	1.61	(1.43 - 1.71)	<0.001
Nationality						
Spanish	1	-	-	1	-	-
Foreign	1.07	(0.96 - 1.18)	0.210	1.11	(0.90 - 1.19)	0.265
Employment situation						
Working	1	-	-	1	-	-
Unemployed	1.81	(1.67 - 1.98)	<0.001	1.33	(1.17 - 1.98)	<0.001
Retired	1.23	(1.12 - 1.35)	<0.001	1.63	(1.60 - 1.75)	<0.001
Studying	1.23	(1.06 - 1.43)	0.070	1.23	(1.16 - 1.43)	0.007
Housekeeping	1.02	(1.14 - 1.35)	0.444	1.84	(1.14 - 1.95)	<0.001
Level of education						
High	1	-	-	1	-	-
Medium	1.36	(1.07 - 1.74)	0.012	1.32	(1.02 - 1.47)	0.012
Low	1.14	(0.88 - 1.46)	0.330	1.14	(0.76 - 1.29)	0.330
Socio-professional class						
Executives and managers	1	-	-	1	-	-
Intermediate occupations, skilled technical, self-employed	1.09	(0.98 - 1.01)	0.050	1.09	(0.98 - 1.01)	0.050
Primary sector skilled workers	1.02	(0.92 - 1.18)	0.385	1.05	(0.94 - 1.37)	0.385
Unskilled workers	1.08	(0.96 - 1.21)	0.200	1.08	(0.96 - 1.21)	0.200
Social support (for 1 point increase)	0.95	(0.93 - 0.98)	<0.001	0.93	(0.92 - 0.97)	<0.001

Among women, widows (OR: 1.40 CI 95%: 1.24-1.57) presented a higher risk of psychic morbidity compared to single women, as did separated or divorced women (OR: 1.61 CI 95%: 1.43-1.71). As regards employment situation, homemakers presented a higher risk of psychic morbidity than working women (OR: 1.84; CI 95%: 1.14-1.95) and retired women (OR: 1.63; CI 95%: 1.60-1.75). Lastly, a link was found between better social support and lower risk of psychic morbidity.

*According to the second multilevel logistic regression model, among the macroeconomic variables studied, those associated with worse mental health for men and women alike were lower healthcare spending per capita and a higher percentage of temporary workers. By contrast, risk of poverty, income per capita per household, Gross Domestic Product and employment rate were not found to be linked to worse mental health* (Table 3)*.*

**Table 3:** Odds Ratio of risk of GHQ ≥ 3 of multi-level logistic regression model with random effects at NUTS-2 regions level according to macroeconomic context variables

Variable	Total			Men			Women		
	OR^a^	95% CI	*p*-value	OR^a^	95% CI	*p*-value	OR	95% CI	*p*-value
Healthcare spending per capita (↓100€ person/year)	1.05	(1.03 - 1.08)	<0.001	1.04	(1.00 - 1.08)	0.046	1.06	(1.03 - 1.09)	<0.001
Risk of poverty (↑10 points)	0.98	(0.89 - 1.08)	0.718	0.96	(0.83 - 1.1)	0.548	0.95	(0.86 - 1.05)	0.357
Income per capita per household (↓20 points)	1.28	(0.86 - 1.89)	0.226	1.08	(0.86 - 1.36)	0.492	1.00	(0.87 - 1.15)	0.996
Gross Domestic Product (↓20 point)	1.05	(0.90 - 1.23)	0.520	0.98	(0.83 - 1.14)	0.767	0.98	(0.87 - 1.1)	0.726
Employment rate (↓5 points)	1.00	(0.96 - 1.03)	0.821	1.02	(0.96 - 1.07)	0.594	0.97	(0.93 - 1.01)	0.140
Unemployment rate (↑5 points)	1.00	(0.99 - 1.02)	0.699	1.02	(0.99 - 1.05)	0.146	0.99	(0.97 - 1.01)	0.323
Percentage of temporary workers (↑5 points)	0.96	(0.94 - 0.99)	0.006	0.92	(0.88 - 0.96)	<0.001	0.99	(0.96 - 1.03)	0.691

(^a^) Adjusted for age, state of health, social class, employment situation, level of education, marital status, nationality and social support

*Among women, the only contextual variable associated to a worse mental health was healthcare spending per capita (the risk of poor mental health increased a 6% for each 100€ decrease in healthcare spending per capita). Among men, the contextual variables associated to a worse mental health were healthcare spending per capita and percentage of temporary workers (the risk of poor mental health decreased 8% for each 5 percentage point increase in temporary workers).*

## Discussion

The severity of the current economic crisis has hit Spain far harder than other European countries, with the possible exceptions of Portugal, Greece and Cyprus [25]. The recession has had a significant impact on conditions and levels of employment and on poverty rates in Spain as a whole, although with considerable differences between Autonomous Communities*. In this respect, in a prior study comparing regions, Zapata states “Spain is currently a natural laboratory for exploring how negative macroeconomic changes affect health”* [25]*.*

*As regards limitations, Parmar* [2] *states that the majority of studies on crises and health are subject to biases, pointing above all to reverse causality or not taking possible prior trends into account. In this study, in the first place,* we have used a short period to study the impact of the crisis with two cut-off points and therefore it is quite possible that mental health has continued getting worse. It was not possible to measure the trend, since in previous years the Health Survey has not measured psychic morbidity. In the second place, *given the cross-sectional nature the possible existence of reverse causality cannot be overlooked. There may be some uncontrolled confusion bias given that other variables are not taken into account (some gathered in surveys and others not) which may or may not have an effect on state of mental health. Yet in s*pite of these limitations, our study is the first of its kind to analyse a multilevel design to investigate the impact of contextual variables during the recession in Spain and its possible consequences on mental health.

The socio-economic factors linked to mental health were healthcare spending per capita and percentage of temporary workers. Estimating the contribution of factors which can affect the health of the population is a complex and inexact task [30]. What does seem clear is that a robust health system can level out inequalities, since it enables support to be given to the most vulnerable sectors of the population [31]. By contrast, a weaker health system (with lower spending) would leave the most vulnerable less protected and these groups are the most exposed in the recession and therefore at higher risk of worse mental health.

Although Spain has a national health system which provides (almost) universal coverage, there is considerable variation in healthcare spending and services from one Autonomous Community to another [32]. It is difficult to find reliable data on healthcare spending specifically for mental health, since budges are not broken down by medical fields. However, it is not unreasonable to believe that it may have suffered the same fate as spending as a whole, at least as regards the most general figures and trends. Inequalities in healthcare spending have a two-pronged effect: a) differences in resource allocation for service provision in different regions (the territorial perspective) and b) differences in public health insurance contributions by individuals or families (the personal perspective) [33]. There is an additional area as regards provision of mental health services which professional associations for mental health have condemned for years: Spain is still bringing up the rear in comparison with other European countries in terms of numbers of mental health practitioners, as shown by official WHO figures [34].

The link between worse mental health and percentage of temporary workers can be understood given that economic recessions can have a direct effect on people who keep their jobs. These individuals face situations of stress and anxiety caused by possible reduction in income, greater employment insecurity and increased workload. Recessions can likewise have a disproportionate negative impact on subgroups in the vulnerable population such as persons with a pre-existing mental disorder, or a low socio-economic level, or the unemployed [35].

*The literature shows contradictory results for the relationship between unemployment and mental health. Some studies have found that unemployment is associated with poorer mental health, particularly amongst women* [36]*, whilst others have found that during recessions or in cases of higher regional unemployment when the number of unemployed people increases and unemployment becomes a status,* the psychological cost and stigma of being unemployed diminishes and the subjective well-being of the unemployed improves [37]. *Taking into consideration the context variables found in our study, these differences would be nuanced by factors such as per capita healthcare spending or percentage of temporary workers.*

In the light of these findings, one might think that different political responses to economic crises would give rise to different mental health outcomes among the population. For example in Spain, unemployment levels in the 70’s and 80’s were accompanied by a corresponding increase in risk of suicide. In Sweden, however, the banking crisis of 1990 left a lot of people unemployed but the suicide rate dropped, even during this period. This marked difference has been attributed to the protection provided by the Swedish welfare state [38, 39].

As regards the measures which should be taken during economic crises to palliate effects on mental health, Kentikelenis and Papanicolas [40, 41] state the need to safeguard programmes for vulnerable groups such as the mentally ill and drug addiction rehabilitation programmes; to increase the number of general practitioners working in rural areas; to taken on the cost of non-medical illnesses among patients; and to prescribe a higher proportion of generic drugs in order to make savings in spending on drugs.

Other studies have highlighted the effectiveness of policies such as active programmes to incentivise the labour market, which have a significant impact on reducing suicide rates [38]. Policies which aim to prevent individuals from taking on too much debt and for making it easier to pay off debts could be beneficial for people whose excessive levels of debt cause them stress [41]. Similarly, policies or initiatives such as financial mediators have huge potential for mitigating the effects of recession [42].

As regards health centres, it has been found that health initiatives for exploring the subjective perception of aloneness can be effective in improving mental health and should focus particularly on individuals in poor health and the unemployed [43]; similarly effective are programmes which support the role of primary care professionals in detecting persons at risk of suicide or other psychological problems [42].

Therefore, instead of making cutbacks in healthcare and social welfare, there should be higher spending on measures for social protection during times of recession and increased support for mental health programmes in the health sector, particularly in primary care [44, 45]. Additionally, there should be more comprehensive and cooperative consolidation of the mental health network within healthcare (social services, primary care, specialised care, and social rehabilitation and reintegration) which takes into account the specific needs of the individuals which this healthcare sector focuses on [45].

## Conclusions

Lastly, data will be required in following years in order to analyse whether fresh government cutbacks to healthcare and social spending [35] and the policies implemented by different Autonomous Communities will have a medium and long-term impact on mental health among the Spanish population. Furthermore, it is to be noted that social inequalities in Spain have increased since the beginning of the financial crisis. Moreover, various studies have highlighted that increased social inequalities are not only an effect of the crisis but also a determining factor of the crisis. Therefore, a more sustainable economic model should make reduction of social inequalities one of its primary goals [46].

### Key points

- Various articles have addressed the impact of socio-economic crises on mental health. They have focused on analysing the effect of individual factors and have left out other factors linked to welfare state public services and economic indicators, which would be proxies for public policies implemented at the regional level.
- The impact of the crisis on the health of the population could be lessened or intensified by policies, affecting the financial security and social conditions of families.
- The findings of this study emphasize that policies during periods of recession should focus on support and improved conditions for vulnerable groups such as temporary workers. Healthcare cutbacks should be avoided in order to prevent increased prevalence of poor mental health among the population.

## Additional file

Additional file 1: Contextual Indicators. (XLSX 12.9 kb)

## Abbreviations

BBVA: Banco Bilbao Vizcaya Argentaria
GDP: Gross domestic product
GHQ: Goldberg health questionnaire
ISCED: International Standard Classification Of Education
NOC: National Occupation Classification
NUTS: Nomenclature des unités territoriales statistiques
